# Design and Dissemination of the *MOVE!* Weight-Management Program for Veterans

**Published:** 2009-06-15

**Authors:** Linda S. Kinsinger, Kenneth R. Jones, Leila Kahwati, Richard Harvey, Mary Burdick, Virginia Zele, Steven J. Yevich

**Affiliations:** VHA National Center for Health Promotion and Disease Prevention; VHA National Center for Health Promotion and Disease Prevention, Durham, North Carolina; VHA National Center for Health Promotion and Disease Prevention, Durham, North Carolina; VHA National Center for Health Promotion and Disease Prevention, Durham, North Carolina; VHA National Center for Health Promotion and Disease Prevention, Durham, North Carolina; VHA National Center for Health Promotion and Disease Prevention, Durham, North Carolina; VHA National Center for Health Promotion and Disease Prevention, Durham, North Carolina

## Abstract

**Background:**

Practitioners in the Veterans Health Administration (VHA) identified comprehensive weight management as a high priority in early 2001.

**Program Design:**

The *MOVE!* Weight-Management Program for Veterans was developed on the basis of published guidelines from the National Institutes of Health and other organizations. Testing of program feasibility occurred at 17 VHA sites, and the program was refined during early implementation throughout 2005.

**Evaluation:**

*MOVE!* has been implemented at nearly all VHA medical centers. By June 2008, more than 100,000 patients had participated in *MOVE!* during more than 500,000 visits. An evaluation based on an established framework is under way.

**Conclusion:**

*MOVE!* is an example of the large-scale translation of research into practice. It has the potential to reduce the burden of disease from obesity and related conditions.

## Background

Overweight and obesity are tremendous health problems for the US population. Recent national prevalence estimates indicate that 66.3% of adults are overweight or obese (1). Similarly, veterans who receive medical care in the Veterans Health Administration (VHA) have a high prevalence of overweight and obesity. A study of 1.8 million veterans who received VHA care in 2000 found a prevalence of overweight/obesity of 73.0% among men (32.9% obese) and 68.4% among women (37.4% obese) (2). According to the 2003 Behavioral Risk Factor Surveillance System, 72.2% of veterans who use the VHA for health care were overweight or obese (3). The reason for the higher prevalence of overweight and obesity among VHA-using veterans is not known. VHA-using veterans, who by definition are seeking medical care, may have different characteristics from people in general community samples, such as those participating in the National Health and Nutrition Examination Survey. Furthermore, VHA users have more physical and mental health conditions than people in community health care settings (4,5). Obesity-associated conditions, such as hypertension, diabetes, ischemic heart disease, and arthritis, are also highly prevalent in the VHA population (6).

Addressing the problem of overweight and obesity in the VHA system is complex. The VHA is the nation's largest integrated health care system; it operated 155 medical centers and 872 community and outpatient clinics in 2006. During that year, the VHA served 5.5 million patients and registered more than 60 million visits within its outpatient clinics (7). Policy and programs are developed centrally then disseminated through the 21 Veterans Integrated Service Networks, within which all VHA medical centers and clinics are organized. At a national VHA preventive medicine meeting in 2001, primary care providers identified effective weight-management interventions as their most pressing preventive medicine need. Informal surveys of VHA medical facilities in 2001 and 2002 showed that, of 106 responding facilities, 42 reported having weight-management programs, varying from individual dietitians' initiatives to, infrequently, comprehensive multidisciplinary programs (unpublished data, National Center for Health Promotion and Disease Prevention, 2001).

## Program Design

In 2002, recognizing the need for a comprehensive, system-wide prevention tool for the VHA, the VHA National Center for Health Promotion and Disease Prevention (NCP) began to develop an evidence-based weight-management program for patients seen at VHA medical facilities. The program incorporated elements essential for gaining support within a large health care system, including an informational letter and other communications to describe for leaders and field-based clinicians the need for such a program and to keep stakeholders aware of the program's development.

The *MOVE!* (Managing Overweight/Obesity for Veterans Everywhere) Weight-Management Program for Veterans (www.move.va.gov) was created on the basis of guidelines from the National Institutes of Health (NIH) (8,9) and other current literature. The program followed the 2003 US Preventive Services Task Force recommendation that "clinicians screen all adult patients for obesity and offer intensive counseling and behavioral interventions to promote sustained weight loss for obese adults" (10).

Designed to be integrated into patients' ongoing care and implemented in primary care clinics, *MOVE!* is a clinical process and set of tools for facilities and clinicians to offer an evidence-based, multidisciplinary, comprehensive approach to weight management that is centered around the patient and tailored to individual needs. All patients seen in primary care clinics are screened at least every 2 years for overweight and obesity using body mass index (BMI). Patients who would benefit from *MOVE!* (those with a BMI ≥30.0 kg/m^2^ or a BMI of 25.0-30.0 kg/m^2^ with 1 or more obesity-related conditions, such as diabetes, hypertension, dyslipidemia, sleep apnea, metabolic syndrome, or arthritis) are assessed for the appropriateness of recommended weight loss (ie, those with limited life expectancy or serious illness are excluded). Patients who indicate readiness to attempt weight loss are offered participation in *MOVE!*. Core treatment uses established behavior change and self-management strategies for diet and physical activity change.

Patients begin participation in *MOVE!* by completing a paper or Web-based 23-item baseline assessment, the *MOVE!23* questionnaire (www.move.va.gov/Move23.asp). This assessment elicits information about medical history; weight and weight-management history; motivational factors; barriers to modifying physical activity, diet, and weight-related behaviors and patients' readiness to change these behaviors. On completion, patients receive a summary report that identifies their individual needs and recommendations, supplemented by tailored links to specific patient handouts. More than 100 handouts about nutrition, physical activity, and behavior change are available in English and Spanish on the *MOVE!* Web site (www.move.va.gov/handouts.asp). The handouts are given to patients by staff or downloaded by patients. The program also produces a staff version of each patient's report, identifying key medical and counseling issues, and automatically enters both the patient and staff reports into the patient's electronic VHA medical record.

After reviewing the *MOVE!23* report with the patient, staff assist the patient with setting 1 to 3 specific short-term nutrition, physical activity, or behavior change goals. Staff provide diet and physical activity logs and may give pedometers to patients to track steps. Staff follow up with patients by telephone or in person in 1 to 3 weeks to determine progress toward or barriers to meeting goals. Ideally, staff-patient contact is ongoing, based on continuing patient need and desire for supported self-management. Many patients also attend group sessions for further instruction and support. Modules for multidisciplinary staff to use in conducting group sessions (www.move.va.gov/GrpSessions.asp) have been developed as part of *MOVE!*. Individual consultation is available for patients who need tailored treatment plans because of comorbid illness or other complicating factors. For patients who need more intensive interventions, Veterans Affairs (VA) facilities provide on a limited basis weight-loss medications, intensive medical management, and bariatric surgery.

## Dissemination

After initial program materials were developed, 17 VHA medical facilities volunteered to participate in pilot feasibility trials between October 2003 and December 2004. Each facility obtained local institutional review board approval and implemented the program using the materials provided — modified to suit local considerations — enrolling 30 to 70 patients for 6 months. To mimic real-world conditions, no additional staff resources or funds were given to sites. Staff and patients evaluated the materials and program for usability and suitability by using surveys, and staff provided feedback during conference calls; overall ratings were positive. Materials were revised and improved in response to staff and patient feedback. The VA Weight Management Executive Council, composed of nationally recognized experts in weight management, nutrition, and physical activity, provided review to aid development. On the basis of pilot results, the council endorsed the program as a state-of-the-art population-based weight-management initiative.

The VA Under Secretary for Health strongly supported the program during all phases of development and system-wide implementation. NCP staff made many presentations at internal VA meetings and external professional meetings. These influences heightened awareness and strengthened demand for the program among clinicians in the field.

Several early adopting medical centers and networks began to fully implement *MOVE!* at their facilities in 2005. During this time, regular conference calls between NCP and staff at these facilities created a forum for sharing successes and challenges related to program implementation and provided an opportunity for input for further program refinements.

*MOVE!* was implemented nationally in January 2006. That month, NCP sent all networks and medical centers a toolkit with starter sets of patient handouts, promotional brochures, clinical references, and administrative manuals (www.move.va.gov/ReferenceTools.asp), as well as marketing materials (eg, posters, banners, pens). Online discipline-specific training modules about weight management with continuing education credit available for physicians, nurses, dietitians, behavioral health specialists, and physical activity specialists were also released.

In March 2006, VHA issued a policy requiring all facilities to implement an evidence-based, comprehensive weight management program and offered *MOVE!* as a model for complying with the new policy (11). Each VA network and facility was required to name a *MOVE!* coordinator and physician champion to implement the program. NCP began twice-monthly conference calls with the network coordinators and has held 2 national training conferences for this group. VHA policy further stipulated that facilities complete an annual report on the status of their weight-management services.

In December 2006, the VA and the Department of Defense (DoD) issued a joint *Clinical Practice Guideline for Screening and Management of Overweight and Obesity* (12). From 2005 through 2007, NCP worked with other VHA offices to develop, disseminate, and provide additional procedures, tools, and guidance to support *MOVE!* implementation, including codes and utilities for tracking *MOVE!*-related visits; sample clinical reminders for the electronic medical record; criteria for use of weight-loss medications; and obesity-related performance indicators. In June 2008, a change in federal regulations eliminated copayments for weight-management clinic visits.

## Evaluation

NCP is conducting a comprehensive mixed-methods evaluation of *MOVE!* using the RE-AIM framework, developed by Glasgow et al (13). This model, designed to evaluate the extent to which research is translated into practice, includes 5 elements: reach, effectiveness, adoption, implementation, and maintenance. Key measures within these elements for the *MOVE!* program are listed in the Table. The evaluation will rely primarily on existing VHA data sources, including the VHA's electronic medical record and *MOVE!* annual report. Additional evaluation questions will require primary data collection (eg, patient surveys to measure changes in physical activity and health-related quality of life, staff surveys to measure attitudes about weight management).

Nearly all (98.7%) facilities now report having *MOVE!* programs in place. Patient participation has steadily increased over time (Figure). Currently, approximately 3,000 to 4,000 patients per month have a first *MOVE!-*related visit. As of June 2008, more than 100,000 patients have had at least 1 *MOVE!*-related visit and 17,699 have had 6 or more *MOVE!*-related visits; more than 500,000 *MOVE!*-related encounters have occurred since tracking began in October 2004. Among all patients enrolled in VA care in 2007, 8.4% were women. During the same timeframe, 12.4% of patients seen with *MOVE!* were women, which suggests that there is greater interest in weight management among female VA patients than among male VA patients.

**Figure 1:**
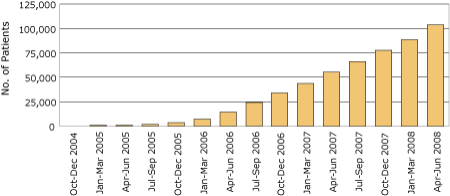
Cumulative number of patients participating in *MOVE!* weight management program, by quarter, US Veterans Health Administration, October 2004 to June 2008.

**Table:** 

**Quarter**	**No. of Patients**
October-December 2004	111
January-March 2005	470
April-June 2005	1,224
July-September 2005	2,182
October-December 2005	3,917
January-March 2006	7,051
April-June 2006	14,446
July-September 2006	24,024
October-December 2006	34,221
January-March 2007	43,797
April-June 2007	54,913
July-September 2007	65,838
October-December 2007	77,580
January-March 2008	88,160
April-June 2008	103,315

Data collected by the VHA's performance measurement system during 2008 indicate that 66% of primary care patients have been screened for obesity. To date, of patients who would benefit from weight management based on BMI, health status, and absence of contraindications, 7.5% have participated in *MOVE!*.

Challenges to this evaluation include the many local modifications to program delivery, which facilitates adoption but introduces heterogeneity, making quantitative analysis difficult. However, with implementation variability comes an opportunity to identify best practices and key elements associated with more effective programs. Clinical data (weight, BMI) extracted from the VHA electronic medical record have not been available nationally until recently, and more validation work is required to be able to accurately interpret these data.

## Discussion

The VHA's *MOVE!* Weight-Management Program has potential for providing evidence-based, comprehensive weight management to the more than 4 million veterans enrolled in VHA health care who are at risk for obesity-related conditions. Created on the basis of a systematic NIH evidence review and designed to be an intensive model of care, as recommended by the US Preventive Services Task Force, *MOVE!* provides an extensive set of program materials for staff and patients to assist with long-term weight management. VHA medical facilities can incorporate this treatment model into local settings in ways that best facilitate their adoption.

Through their collaborative work (12), VA and DoD practitioners have shared their weight-management tools and resources. A recently released toolkit for providers in both systems will incorporate most of the *MOVE!* tools that have been developed, which will extend the reach of *MOVE!* to active duty members and their families. In 2007, the DoD Weight Management and Education Workgroup was charged with developing an evidence-based program of care that could be implemented in DoD medical treatment facilities and has adopted *MOVE!* as the standard weight-management program if weight-management programs are not in place.

The program has also been extended to VA employees. Soon after implementing *MOVE!* for patients, some facilities began to use *MOVE!* materials and procedures for the benefit of VA employees. A *MOVEmployee!* manual, describing alternative ways to implement these services, was added to the program materials in 2006. As of late 2007, 63 employee programs existed.

Another opportunity to extend the reach of *MOVE!* has been through the HealthierUS Veterans initiative, a collaboration between the VA and Department of Health and Human Services (14). HealthierUS Veterans is a health promotion campaign aimed at reaching the larger community of veterans (including those not seen at VA facilities) and their family members with messages about eating healthfully, being physically active, and staying "fit for life." One component of the HealthierUS Veterans campaign is to promote the use of the online *MOVE!* resources, including the *MOVE!23* and patient handouts, outside of the VHA. Veterans and family members may complete the assessment, print out both the individual and staff reports and associated handouts, and take them to their non-VHA medical providers.

*MOVE!* implementation has helped to raise the profile of obesity research within the VHA community. A number of VA- and NIH-funded studies have been completed or are in progress and include an investigation of changes in obesity care practices within VHA; a study comparing enhanced physical activity interventions with *MOVE!*; studies adapting *MOVE!* materials for special populations, including veterans with serious mental illness and veterans with spinal cord injuries or disease; a study of the long-term outcomes of bariatric surgery within the VHA; and a study evaluating 2 enhancements to usual *MOVE!*-tailored patient newsletters and peer-led self-management support.

The NCP continues to develop and refine *MOVE!*. Many veterans live at considerable distances from VHA medical centers or community clinics, so plans include enhancing Web-based tools to be more interactive. VHA has an extensive program for home telehealth management of chronic diseases, such as diabetes and heart failure. Development of a *MOVE!* telehealth program for overweight and obese patients with metabolic syndrome or prediabetes is under way. This program will encourage patients to weigh themselves frequently and use a simple electronic device to report eating and physical activity patterns. Using the device will provide encouragement and support for patients who meet their goals and will alert staff when monitored parameters become out of range. To facilitate ongoing telephone contact between patients and staff for self-management support, plans for regional call centers with health coaches are being developed.

The VA's *MOVE!* Weight-Management Program for Veterans is an innovative and comprehensive strategy to provide sophisticated treatment for overweight and obesity to veterans. The program also provides an opportunity to study the best strategies for delivering this care and for measuring the health outcomes of patients who receive it. Similar to VHA's implementation of quality improvement and system change for tobacco control (15), *MOVE!* is an example of translation of research and evidence-based medicine into broad medical practice in a large health care organization.

## Acknowledgments

Partial funding for initial development of *MOVE!* resources was provided by Roche through an unrestricted educational grant to the VA. All other funding has been provided by the VA. The views expressed in this article are those of the authors and do not necessarily represent the views of the VA.

## Figures and Tables

**Table.** Select Measures for the *MOVE!* Program Evaluation^a^, *MOVE!* Weight-Management Program for Veterans, United States, 2008

**Reach**
Percentage of VHA patients screened for overweight or obesity and offered *MOVE!* if applicable Percentage of overweight or obese patients receiving weight-management treatment Percentage of facilities with 10% or more of their overweight/obese population receiving treatment
**Effectiveness**
Average percent body weight and BMI change of participants at 6 months, 1 year, and beyond Percentage of participants who have lost ≥5% of body weight at 6 months, 1 year, and beyond Percentage of participants who maintained weight loss at various time intervals after participation
**Adoption**
Staff time and effort spent coordinating and providing *MOVE!* at each facility Percentage of facilities with distinct *MOVE!* budgets and formal service agreements Percentage of facilities that provided regular weight-management education and/or training to staff
**Implementation**
Percentage of facilities that use a multidisciplinary approach that includes staff from all key disciplines (dietetics, physical activity, behavior, and primary care) and percentage providing behavioral counseling and support in both nutrition and physical activity Percentage of facilities using specific program elements (eg, electronic screening, clinical reminder, individual self-management support, group sessions, individual specialty consultation, weight loss medications, intensive behavioral treatment, bariatric surgery) Average intensity of treatment (number of contacts per patient treated)
**Maintenance**
Barriers to and facilitators of program adoption and maintenance

Abbreviations: VHA, Veterans Health Administration; BMI, body mass index.

a Based on the RE-AIM Framework of Glasgow et al (13).
